# Transmission of Hepatitis C Virus among Prisoners, Australia, 2005–2012

**DOI:** 10.3201/eid2105.141832

**Published:** 2015-05

**Authors:** Neil Arvin Bretaña, Lies Boelen, Rowena Bull, Suzy Teutsch, Peter A. White, Andrew R. Lloyd, Fabio Luciani, on behalf of the HITS-p investigators

**Affiliations:** The University of New South Wales, Sydney, New South Wales, Australia (N.A. Bretaña, L. Boelen, R. Bull, S. Teutsch, P.A. White, A.R. Lloyd, F. Luciani);; Imperial College London, London, UK (L. Boelen)

**Keywords:** hepatitis C virus, transmission, phylogenetics, clustering, injection drug use, prisons, viruses, Australia

## Abstract

Hepatitis C virus (HCV) is predominantly transmitted between persons who inject drugs. For this population, global prevalence of HCV infection is high and incarceration is common and an independent risk factor for HCV acquisition. To explore HCV transmission dynamics in incarcerated populations, we integrated virus sequences with risk behavior and spatiotemporal data and analyzed transmission clusters among prisoners in Australia. We detected 3 clusters of recent HCV transmission consisting of 4 likely in-custody transmission events involving source/recipient pairs located in the same prison at the same time. Of these 4 events, 3 were associated with drug injecting and equipment sharing. Despite a large population of prisoners with chronic HCV, recent transmission events were identified in the prison setting. This ongoing HCV transmission among high-risk prisoners argues for expansion of prevention programs to reduce HCV transmission in prisons.

## Abstract 2

Ongoing transmission is associated with drug injection.

Hepatitis C virus (HCV) is a blood-borne virus that infects 3–4 million persons each year (*1*). In industrialized countries, transmission of HCV is largely attributed to injection drug use (*2*). The association between injection drug use, HCV infection, and imprisonment is very close (*3*). People who inject drugs (PWID) account for a large proportion of the incarcerated population in the United States, Canada, Europe, and Australia (*4*–*7*), and injection drug use is prevalent during incarceration (*8*,*9*). Globally, the prevalence of HCV infection among prisoners is ≈30% (*10*,*11*). A meta-analysis of 30 studies conducted in different countries revealed a clear association between the prevalence of HCV infection among prisoners and a history of injection drug use (*6*).

A recent meta-analysis of HCV incidence studies among prisoners revealed a mean incidence of 16.4 (95% CI 0.8–32.1) cases per 100 person-years (*11*). We recently documented incidence of 14.1 (95% CI 10.0–19.3) cases per 100 person-years in 37 prisons in New South Wales (NSW), Australia, and identified recent injection drug use and Aboriginal and Torre Strait Islander descent as independent risk factors for HCV seroconversion (*12*). This analysis also identified high prevalence of injection drug use and sharing of injecting equipment in prisons (*12*). Furthermore, 13 incident cases were identified in a subcohort of 114 prisoners continuously imprisoned (i.e., without release to the community) during the study period (incidence 10.3 cases/100 person-years).

Prisons can be regarded as an enclosed network of facilities within which prisoners are frequently moved. In NSW, prisoners are often transferred between prisons (e.g., because of changes in prisoner security classifications) and temporarily moved for brief periods (e.g., to go to court or obtain medical treatment). In addition, prison sentences in Australia are typically short (average 7–9 months), but reincarceration rates are high (*13*).

The HCV genome evolves rapidly by mutations caused by highly error-prone replication mechanisms, which generate a swarm of constantly evolving variants (quasispecies) during every infection (*14*). HCV is classified into 7 genotypes and 67 subtypes (*15*). At the nucleotide level, each virus subtype differs by up to 25% and genotypes differ by up to 33% (*16*). The hypervariable region (HVR) of the HCV genome is the most variable; hence, this region is commonly used in molecular epidemiologic studies to detect clusters of persons infected via recent transmission events (*17*). We used sequences covering envelope (E) 1 and partial E2 (HVR1). 

Acute HCV infection is largely asymptomatic; hence, the precise timing and source of transmission are usually unknown. Accordingly, virus sequencing and phylogenetic analysis have been used to reconstruct probable transmission chains from prevalent cases (*18*–*20*). Although broad linkages between HCV-infected persons have been demonstrated, previous efforts to identify probable transmission pairs among infected persons by using a combination of social network information and phylogenetic analysis techniques suggested that social and genetic distances were only weakly associated (*21*). By contrast, a recent report from a study that used this same approach among both prevalent and incident (newly infected) case-patients, identified probable clusters evidenced by proximity of social network and clustering analysis of core HCV sequences in a community-based cohort of PWID (*22*).

Our study used an integrated analysis of molecular, epidemiologic, and spatiotemporal data from a well-characterized cohort of longitudinally followed PWID. We used incident case detection in prisons to identify clusters of recent HCV transmission.

## Methods

### Hepatitis C Incidence and Transmission Study 

The Hepatitis C Incidence and Transmission Study in Prisons (HITS-p) is a prospective study of a cohort of 498 prisoners with a history of injection drug use recruited from 37 prisons in NSW during 2005–2012 (*12*,*23*,*24*). At the time of preenrollment screening, all HITS-p participants were not infected with HCV; 181 subsequently became infected (*12*,*23*,*24*).

### Study Cohort

For our study, we considered a HITS-p subset of 79 prisoners infected with HCV genotype 1 or genotype 3 for which HCV E1-HVR1 sequences were available. At ≈6-month intervals during participants’ incarceration, we collected demographic information, lifetime and follow-up risk behavior data, and blood samples for HCV serologic and virologic testing (*12*,*23*,*24*). These data were collected by a trained research nurse whose employment was independent of the prison system (*12*).

### HCV Testing and Estimated Date of Infection

Blood samples were tested for presence of HCV RNA and antibodies as described elsewhere (*12*,*23*,*24*). For participants who had seroconverted at the incident time point (the time of sampling when a person is found to have already seroconverted), the date of infection was estimated as the midpoint between the first HCV antibody–positive and the last HCV antibody–negative test result. For participants who were HCV RNA positive but HCV antibody negative at the incident time point, the date of infection was estimated to be 51 days before the date of sampling (*25*).

### Statistical Analyses

We used *t*-tests (for continuous variables) and χ^2^ tests (for categorical variables) to compare the demographic characteristics and risk behavior of newly infected participants with those of noninfected participants (significance level = 0.05). We used the Wilcoxon rank-sum test to assess differences in number of movements.

### Sequencing of the E1-HVR1 

The region encoding the last 171 bp of core, E1, and HVR1 (882 bp [nt 723–1604]) was compared with HCV strain H77 (GenBank accession no. AF009606). These sequences were then amplified by nested reverse transcription PCR as described elsewhere (*26*). 

### Phylogenetic Analysis

ClustalW (implemented in MEGA 5.2.1 [*27*]) was used for alignment of genotypes 1 and 3 E1-HVR1 sequences. Alignments were visually inspected and manually edited. The HKY model with gamma distribution and a proportion of invariable sites was selected as the best-fit evolutionary model by using JModelTest (*28*). Separate phylogenetic trees for the genotype 1 and genotype 3 alignments with a maximum-likelihood approach were generated by using PhyML (*29*). To check for the robustness of the trees, we performed a 1,000-bootstrap test.

### Clustering Analyses

Clusters of recent HCV transmission were detected by using PhyloPart (*30*), a software program that identifies genetically related sequences from a given tree by use of a statistical algorithm based on analysis of pairwise patristic distances (the amount of change between any 2 sequences as depicted by the branch lengths in a phylogenetic tree). PhyloPart considers any subtree as a cluster if the median pairwise patristic distance among its members is below a set percentile threshold of the distribution of all pairwise patristic distances in the given tree (Technical Appendix).

### Validation Analyses of Clusters of Recent HCV Transmission

Records for each participant (consisting of time, date, and location of entry and exit from each prison) during 2005–2012 were obtained from the NSW Department of Corrective Services. Recent HCV transmission events were validated by integrating the estimated date of infection, incarceration time and location, and the reported risk behavior of participants during follow-up in each of the phylogenetically designated clusters.

For each cluster of cases indicating recent transmission, potential transmission pairs (source and recipient) are identified as any 2 participants co-located in the same prison for at least 24 hours. The source was identified as the participant with an estimated date of infection earlier than the time of co-location with the other participant. The recipient was identified as the participant who was HCV antibody negative before co-location and who became HCV antibody positive within 12 months after co-location with the source participant. Clusters of >2 participants were considered valid with the identification of at least 1 transmission pair.

Risk behaviors (assessed prospectively during interviews at 6-month intervals) were available for the HITS-p cohort and included injection drug use and other blood-to-blood contact but excluded risks associated with sexual behavior (*12*). Information about drug injection and sharing of injecting equipment were obtained “since coming into prison” or “since the last interview” in association with “injected drugs,” “frequency of injecting drugs,” “use of injecting equipment after someone else,” and “frequency of use of injecting equipment.” 

## Results

### Participants

From 181 newly infected participants (incident case-participants) in the HIT-P cohort, 102 were excluded from the study because they were infected with an HCV genotype other than 1 or 3. The study cohort thus comprised 79 viremic incident case-participants. Most (49 [62%]) participants were male, mean ± SD age was 28 ± 7.2 years, 18 (23%) were of Aboriginal and Torre Strait Islander descent, and 61 (77%) had completed <10 years of formal education. The study cohort included 69 (87%) participants who had been previously imprisoned, and most had lifetime risk factors for blood-borne virus acquisition at baseline (Table 1). No significant differences in demographics and lifetime risk behaviors were found between the 79 study cohort participants and the 317 noninfected HITS-p cohort participants, other than previous imprisonment and having ever injected drugs while in prison (Table 1). There were no significant differences between the 79 study cohort participants and the 102 excluded infected participants (Table 1).

**Table 1:** Demographic characteristics and lifetime risk behavior of prisoners in New South Wales, Australia, 2005–2012*

Characteristic	Infected prisoners/ study cohort, n = 79†	Noninfected prisoners, n = 317	p value‡	Infected prisoners excluded, n = 102§	p value¶
Mean (± SD) age, y	28 (7.2)	28 (7.0)	0.71	26 (6.5)	0.13
Median (± SD) time since initiation of injecting, y	6.5 (6.3)	7 (6.3)	0.81	7 (6.1)	0.60
Male sex	49 (62)	216 (68)	0.41	60 (59)	0.78
Aboriginal and/or Torres Strait Islander	18 (23)	58 (18)	0.44	37 (36)	0.07
>10 y of education	61 (77)	238 (75)	0.73	84 (82)	0.50
Previously imprisoned	69 (87)	215 (68)	**0.001**	77 (75)	0.07
Ever had a tattoo	58 (73)	228 (72)	0.84	74 (73)	1
Ever injected drugs in prison	26 (33)	67 (21)	**0.04**	42 (41)	0.33
Ever shared injecting equipment in prison	23 (29)	61 (19)	0.06	37 (36)	0.43

*Data are expressed as no. (%) unless otherwise indicated. HITS-p, Hepatitis C Incidence and Transmission Study in Prisons. †Study cohort = viremic participants from the HITS-p cohort. ‡2-sided comparison of participants from the study cohort and noninfected participants from the HITS-p cohort. §102 prisoners were excluded because they were infected with an HCV genotype other than 1 or 3. ¶2-sided comparison of participants from the study cohort and infected participants excluded from the study.

### Phylogenetics

**Figure 1:**
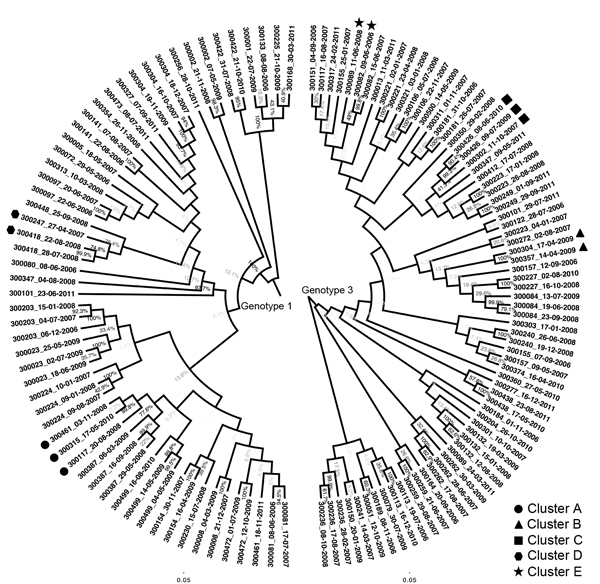
Phylogenetic trees composed of 129 sequences from 79 participants infected with hepatitis C virus genotypes (gt) 1a, 1b, or 3a, New South Wales, Australia, 2005–2012. Names on the tips of the tree represent participant identification numbers and are followed by the sample collection date. Each phylogenetic tree was generated separately from a maximum-likelihood model by using an HKY substitution model with gamma distribution. Bootstrap values are >80% for all branches of identified transmission clusters. Bootstrap values between branches representing sequences from the same host were lower than those between host branches. Identified transmission clusters are labeled with symbols. Scale bars indicate nucleotide substitutions per site.

A total of 129 sequences of E1-HVR1 were obtained from the 79 participants; 26 participants were infected with HCV genotype 1a, 5 with genotype 1b, 44 with HCV genotype 3a, and 4 with HCV genotypes 1a and 3a at different times. These reinfection cases were included in both the genotype 1 and genotype 3 analyses with the corresponding genotype-specific sequences. For participants infected with genotype 1, sequences were available from 1 viremic time point for 19 participants, from 2 time points for 10, and from 3 time points for 6. For participants infected with genotype 3, sequences were available from 1 viremic time point for 28 participants, from 2 time points for 15, and from 3 time points for 5. Phylogenetic trees were constructed for the genotype 1 and genotype 3 E1-HVR1 sequences (Figure 1).

### Clustering 

**Figure 2:**
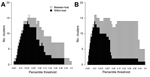
Analysis of hepatitis C virus transmission clusters identified across a range of percentile thresholds among prisoners in New South Wales, Australia, 2005–2012. Analysis shows the relationship between the number of clusters detected and the percentile thresholds from the distribution of genetic distances generated by using genotype 1 (A) and genotype 3 (B) sequences. At the lowest percentile threshold, only clusters containing sequences from the same participant are detected (black bars). When this threshold is increased, clusters of sequences from distinct participants arise (white bars).

The optimal cutoff patristic distance designating recent transmission clusters was determined first by investigation of a range of percentile thresholds from the distribution of pairwise patristic distances (Technical Appendix Methods). As expected at the minimum percentile value, only within-participant clusters were detected, while at the maximum, all sequences for each genotype were included in a single between-participant cluster (Figure 2). On this basis, the chosen cutoff patristic distance for designation of between-participant clusters was 0.099 for genotype 1 and 0.095 for genotype 3 (corresponding to 0.034 and 0.022 nt substitutions/site in the E1-HVR1 region, respectively).

**Figure 3:**
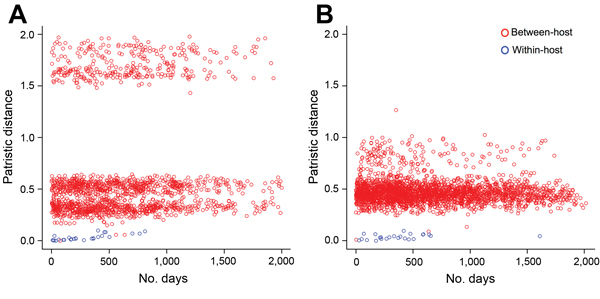
Analysis of pairwise patristic distances between hepatitis C virus sequences from the same participant (within-participant) sampled over time, and from between participants also sampled over time, among prisoners in New South Wales, Australia, 2005–2012. Analysis shows pairwise patristic distances as a function of the time interval between 2 sampling time points: within-participants (blue circles) and between-participants (red circles) for genotypes 1 (A) and 3 (B). A) Blue circles represent data from 35 participants, for a total of 57 sequences; B) blue circles represent data from 49 participants, for a total of 73 sequences.

To assess the effect of the time interval between sampling points on the distribution of pairwise patristic distances, and hence the designated thresholds, we studied the relationship between the time of collection and the pairwise patristic distance between all the sequences available for the study cohort (longitudinally within-participant and between-participant). The pairwise patristic distances between hosts was independent of the time interval (Figure 3). The degree of viral divergence reflected by patristic distances among sequences from within the same participant increased with the time interval between the collection time points. Within the time window analyzed (up to 4 years), within-participant genetic distances remained smaller than those from between-participant pairs. Only a small proportion of the between-participant genetic distances were within the range of within-participant pairs.

Further validation analyses including sequences from a single-source HCV outbreak (Technical Appendix Results 1) showed that within-participant evolution could generate patristic distances greater than those observed between the sequence of the source and infected recipients when collected up to 23 years after transmission. However, the median distribution of these distances revealed that between-participant distances were significantly higher than within-participant differences.

Last, to assess the potential effect of virus diversity within the quasispecies of a single-source host and the potential transmission of a minor variant to a new recipient, the distribution of pairwise patristic distances between all E1-HVR1 variants within the quasispecies from 2 time points collected over 1 year from 2 participants followed from primary HCV infection was analyzed to a sensitivity of variants representing 1% of the quasispecies (Technical Appendix Results 2). Again, the maximum within-participant genetic distance within the quasispecies did not exceed the genetic distances between consensus sequences identified in between-participant analyses.

### Clusters of Recent Transmission and Spatiotemporal Validation

One cluster of recent transmission was detected among 57 genotype 1 sequences (Figure 1, cluster A). This cluster consisted of 3 participants (nos. 117, 461, and 315); median pairwise patristic distance was 0.058. Two clusters were detected among genotype 3 sequences. The first (Figure 1, cluster B) consisted of 2 participants (nos. 304 and 357); median pairwise patristic distance was 0.011. The second cluster (Figure 1, cluster C) consisted of 2 participants (nos. 426 and 302); median pairwise patristic distance was 0.090. Two more clusters were detected just above the designated patristic distance cutoff (Technical Appendix Results 3). The estimated date of infection, incarceration time and location, and reported risk behavior for each cluster member were analyzed to provide convergent evidence for likely transmission events (Table 2).

**Table 2:** Probable HCV transmission events identified by using phylogenetic analysis, spatiotemporal information, and risk behavior information, New South Wales, Australia, 2005–2012*

Cluster	Transmission, participant ID no.	Period of co-location	Prison ID†	Patient ID	Est. date of infection	HCV genotype	ATSI	Continuously in prison‡	Equipment sharing§	OST§	Heroin use§
A	315 → 117	2007 Dec 31–2008 Jan 22	AT	315	2007 Oct 30	1a	No	Yes	Yes	No	No
				117	2008 Feb 27	1a	No	No	Yes	No	Yes
	315 → 461	2008 Jun 29–Jul 11	AE	315	2007 Oct 30	1a	No	Yes	Yes	No	No
		2008 Sep 24–Oct 1		461	2008 Oct 6	1a	No	No	Yes	No	Yes
B	304 → 357	2007 Oct 26–Nov 23	AB	304	2007 Apr 17	3a	No	No	No	No	No
				357	2008 Nov 11	3a	No	Yes	Yes	No	Yes
C	302 → 426	2008 Dec 9–18	AP	302§	2007 Oct 11	3a	Yes	No	Yes	No	No
				426	2008 Dec 21	3a	Yes	No	Yes	Yes	No

*All prisoners were injection drug users during the period of co-location. ATSI, Aboriginal and/or Torres Strait Islander descent; est., estimated; HCV, hepatitis C virus; ID, identification; OST, opioid substitution therapy. †Prisons are identified by codes for de-identification purposes. ‡Continuously in prison 6 mo before estimated date of infection. §Female patient. All others were male.

**Figure 4:**
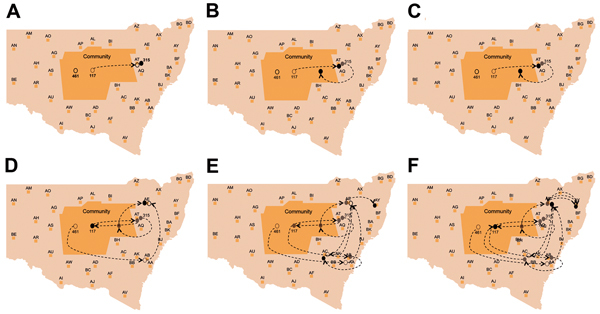
Reconstruction of the likely hepatitis C virus transmission dynamics among prisoners in New South Wales, Australia, 2005–2012**.** Geographic representation of the transmission dynamics among 3 participants identified in cluster A over a 12-month period and co-location dynamics of these participants during October 2007–October 2008 between the prisons in New South Wales are shown. Participants moved between 4 prisons and between prisons and the outside community (arbitrarily located in the center of the map of New South Wales). A) Time 0 (earliest record of location of the cluster members before co-location events occurred between any of the pairs within the cluster); B) 2 months after time 0; C) 4 months after time 0; D) 8 months after time 0. E) 10 months after 0, F) 12 months after time 0. Prisons are de-identified, indicated with a 2-letter code and random locations. Arrows represent the movement of participants between 2 prisons. Filled ovals indicate viremic participants; empty ovals indicate nonviremic patients; gray indicates previous location (past movements) of each participant.

These dynamic participant movements were reconstructed for each transmission cluster. In cluster A, HCV was likely to have been transmitted from participant 315 to participants 117 and 461 (Figure 4). The estimated date of infection with genotype 1a for participant 315 was October 30, 2007; this participant had been in the same prison as participant 117 for 22 days (December 31, 2007–January 22, 2008). Both participants reported injecting drugs and sharing injecting equipment during the period of co-location. Participant 117 was then found to be viremic with genotype 1a in a sample obtained on August 20, 2008, giving an estimated date of infection of February 27, 2008. In another likely transmission event, participant 315 had been in the same prison with participant 461 on 2 occasions: for 13 days (June 29–July 11, 2008) and for 9 days (September 24–October 1, 2008). Both participants reported injecting drugs and sharing injecting equipment during the period of co-location. Participant 461 was then found to be viremic with genotype 1a according to a sample dated November 3, 2008; estimated date of infection was October 6, 2008 (Video 1, http://wwwnc.cdc.gov/EID/article/21/5/14-1832-F1.htm). In transmission cluster B, HCV was likely to have been transmitted from participant 304 to 357. Estimated date of infection with genotype 3 for participant 304 was March 17, 2007; this participant had been in the same prison with participant 357 for 28 days, October 26–November 23, 2007. Both participants reported injecting drugs (although participant 304 did not report sharing injecting equipment) during the period of co-location. Participant 357 was then found to be viremic with genotype 3 according to a sample dated April 17, 2009; estimated date of infection was September 11, 2008 (Video 2, http://wwwnc.cdc.gov/EID/article/21/5/14-1832-F2.htm). In transmission cluster C, HCV genotype 3 was likely to have been transmitted from participant 302 to participant 426. Estimated date of infection for participant 302 was October 11, 2007; this participant had been in the same prison with participant 426 for 9 days, December 9–18, 2008. Both participants reported injecting drugs and sharing injecting equipment during this period of co-location. Participant 426 was then found to be viremic according to a sample obtained on July 9, 2009; estimated date of infection was December 21, 2008 (Video 3, http://wwwnc.cdc.gov/EID/article/21/5/14-1832-F3.htm). Of note, participant 302 is female, and participant 426 is male. Despite the short period of co-location, it is unlikely that prisoners of different sex could interact directly in the prisons, although shared use of a single injection device may have been possible.

**Video 1:**
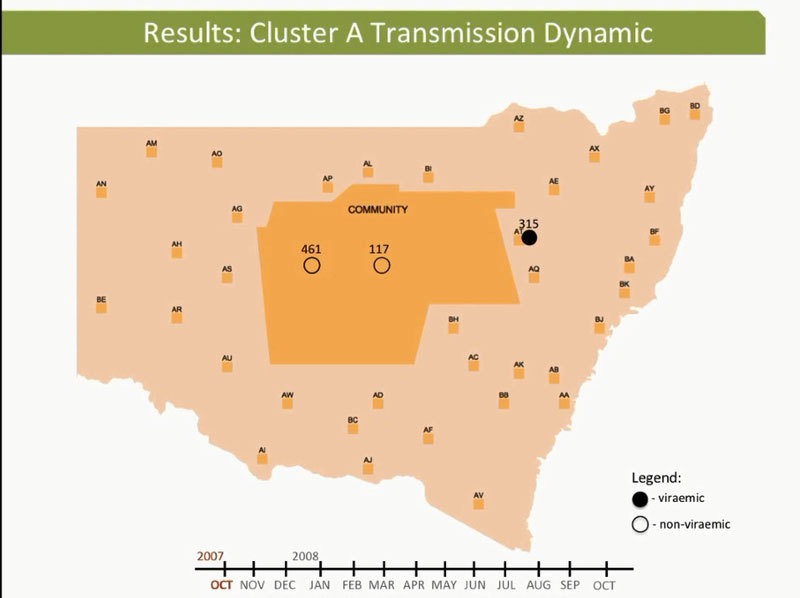
Hepatitis C virus transmission dynamics among prisoners in cluster A, involving participants 315, 117, and 461, New South Wales, Australia, 2005–2012.

**Video 2:**
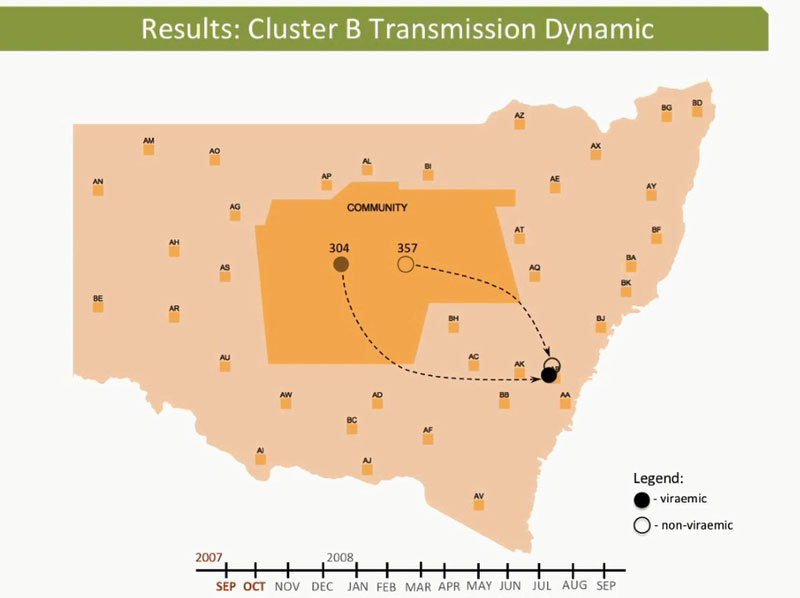
Hepatitis C virus transmission dynamics among prisoners in cluster B, involving participants 304 and 357, New South Wales, Australia, 2005–2012.

**Video 3:**
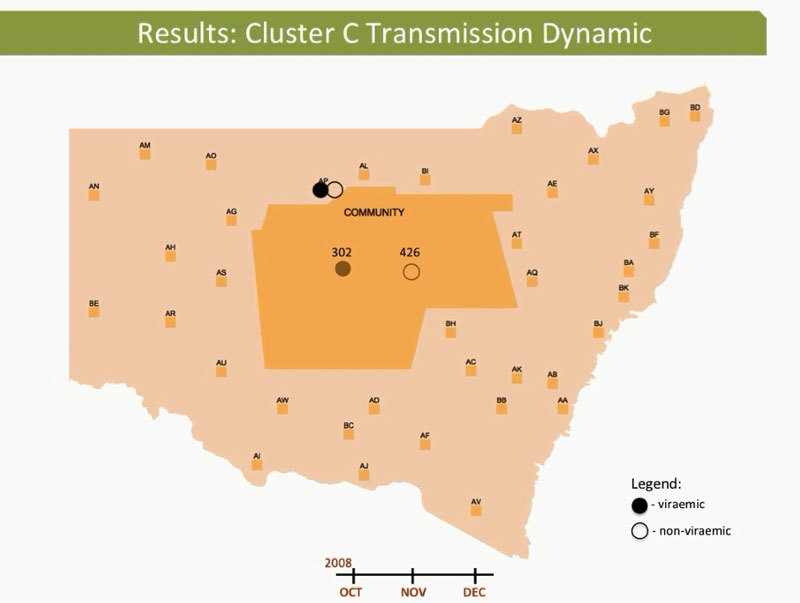
Hepatitis C virus transmission dynamics among prisoners in cluster C, involving participants 302 and 426, New South Wales, Australia, 2005–2012.

### Relationship between Phylogenetic Clustering and Movement Dynamics

In NSW, a high number of prisoner movements are common; prisoners are often transferred between correctional centers or released to the outside community. During the study period (2005–2012), participants from the HITS-p cohort were moved to a different location (a prison or the outside community) a mean of 17 times (Technical Appendix Table 2), and the 79 participants in the study cohort moved a mean (± SD) of 22 ± 13.55 times, with a mean of 4 ± 2.83 release events**.** The 7 participants from the 3 clusters of recent HCV transmission moved to a different location a mean of 28 ± 15.75 times, a significantly greater number of times than for the HITS-p cohort as a whole (p = 0.002) and for the subcohort of uninfected participants (p<0.001). These differences remained significant when movements from one prison to another and release to outside community were tested separately (p<0.05 for all).

## Discussion

Our molecular epidemiology analysis combined with detailed spatiotemporal and behavioral risk data identified several clusters of recent transmission of HCV infection within NSW prisons. This study shows direct evidence of ongoing HCV transmission among PWID in a prison setting.

Previous phylogenetic studies have examined associations between HCV infection and risk and demographic characteristics, including injection drug use (*17*,*21*,*22*,*31*,*32*). Moreover, those studies have defined transmission clusters with a threshold value fixed a priori, such as a maximum genetic distance of 2%–5% (*17*), or with a bootstrap cutoff value (*22*). Here, an empirically optimized threshold, which can also be larger than the typical threshold fixed in previous studies, was used to search for clusters of recent transmission exclusively among incident case-participants.

Despite a high prevalence of chronic HCV infection in prison populations, 3 clusters of transmission were identified in phylogenetic analysis of only 79 participants with recent HCV infection identified during 2005–2012. During this period, ≈20,000 persons were imprisoned annually in NSW; HCV antibody prevalence was ≈30% (*33*,*34*), which equates to ≈4,500 persons with chronic HCV infection (assuming 25% of those cleared infection) who were imprisoned annually. When discounted for 40% recidivism (*13*), this calculation yields ≈19,000 infected prisoners who may have acted as sources for HCV transmission over the study period. In our analysis, the numbers of movements were higher among newly infected participants than among noninfected participants, suggesting that transmission is associated with frequent movements between prisons and from prison to the outside community. Such frequent movements could increase the chance of contact with infected persons or could be otherwise associated with behavior that puts a person at increased risk for HCV transmission.

It is possible that recently infected participants are more likely than chronically infected participants to transmit infection (*35*). This possibility could result from higher infectivity of the transmitted founder viruses, which are intrinsically adapted for successful transmission and dominate the acute phase of infection (*14*). In contrast, a high circulating viral load is associated with an increased probability of vertical HCV transmission (*36*,*37*). However, in our study of PWID, the viral loads (recorded in the blood samples close to the time of transmission) in the source case-participants in the clusters were only low to moderate (data not shown). An alternative explanation is the possibility that these clusters are part of an existing network of high-risk PWID across prisons.

The genetic diversity between variants within the quasispecies during a single infection can become substantial because of the high mutation rate of the virus and the selection pressures of the host immune response. This diversity could influence transmission events because a minor variant in the source can be preferentially transmitted and then dominate the virus population in the recipient host. Therefore, consensus sequencing might not be sufficient for detection of clusters in which transmission is driven by rare variants. Despite the fact that the maximum genetic distances observed within the quasispecies in the selected samples studied here did not exceed the mean genetic distance between hosts, it remains possible that additional transmission clusters may have become evident had this approach been used for all samples.

Our study has several limitations. First, the virus populations involved in transmission events occurring several months after infection might differ from those involved in the acute phase of infection because of the rapid diversification of the virus genome. Therefore, these findings may underestimate ongoing transmission in prisons. Second, although the viruses infecting persons in the clusters were closely related, there is a possibility that unknown participants outside the cohort were also part of the transmission chains; hence, the identified recipient could have been infected by an intermediary source. This possibility may be relevant to probable indirect transmission of HCV from a female participant to a male participant in cluster C because male and female prisoners are segregated in prisons in Australia. Third, because the proposed method uses information collected only during incarceration, data on injecting and sharing behavior in the outside community were not available. Indeed, only 20 (25%) prisoners in the study cohort were continuously imprisoned in the 6 months before the estimated date of infection. Finally, risk behavior could have been underestimated because of the underreporting of sensitive and socially stigmatized behavior during interviews.

From a global perspective, public health control programs have had relatively limited effects on mitigating HCV transmission. The analysis of the HITS-p cohort showed that opioid substitution therapy uptake reaches only 20% of the population (*12*,*24*), despite 64% reporting having ever injected heroin. A recent study on a cohort of PWID in NSW has identified a strong protective effect of opioid substitution therapy (*38*). The combination of needle and syringe exchange programs and opioid substitution therapy programs is the most effective approach for mitigating HCV transmission, reducing incidence by a substantial amount (30%–80%) (*39*,*40*). However, needle and syringe exchange programs remain prohibited in NSW prisons. By identifying ongoing HCV transmission in prisons, this study advocates for new strategies for reducing risk behavior, such as increasing opioid substitution therapy use and eventually introducing needle and syringe programs in prison settings.

Technical Appendix Supplementary methods and results for study of transmission of hepatitis C virus among prisoners, Australia, 2005–2012
