# Supplementary material for: Transmission of Hepatitis C Virus among Prisoners, Australia, 2005–2012
Source: Emerg Infect Dis. 2015 May;21(5):765–74. doi: 10.3201/eid2105.141832 (PMC4414091; doi:10.3201/eid2105.141832)
Supplement: Technical Appendix — Supplementary methods and results for study of transmission of hepatitis C virus among prisoners, Australia, 2005–2012 [file 14-1832-Techapp-s1.pdf]

Article DOI: http://dx.doi.org/10.3201/eid2105.141832

Transmission of Hepatitis C Virus among Prisoners, Australia, 2005–2012

Technical Appendix

Methods

Algorithm for the detection of a recent transmission cluster based on pairwise patristic distance analysis.

Clusters of recent HCV transmission were detected using PhyloPart (*1*). This software detects clusters of genetically-related sequences from a given tree using a statistical algorithm based on an analysis of pairwise patristic distances which correspond to the amount of genetic change between any two sequences as depicted by the branch lengths in a phylogenetic tree (*2*). PhyloPart detects any sub-tree as a cluster, if the median pairwise patristic distance among its members is below a set percentile threshold. The percentile threshold is an adjustable parameter that is defined as the nth percentile of the whole-tree pairwise patristic distance distribution.

For this analysis, the following algorithm was applied separately on gt1 and gt3 trees to detect clusters of recent HCV transmission:

1. Consider a phylogenetic tree of E1-HVR1 sequences.
2. From the distribution of clusters estimated via PhyloPart, identify the range of percentile thresholds, which allows detection of clusters containing sequences from 2 or more subjects, regarded as potential between-host clusters.
3. Define the minimum percentile threshold as that for which only clusters containing sequences from the same subject (within-host clusters) are detected. Also, define the maximum percentile threshold as the value at which all sequences constitute a single between-host cluster.
4. Identify empirically a cut-off pairwise patristic distance defined as the maximum pairwise patristic distance from longitudinal within-host sequences in the analysis

cohort. In order to do this, the evolution between pairs of sequences as shown by pairwise patristic distances over time in longitudinal samples was considered.

5. Identify a clustering threshold by implementing a search algorithm starting from the identified minimum percentile threshold value and increasing its value by 0.001. For each incremental step, consider the median pairwise patristic distance of each between-host cluster detected. If all the median patristic distances of the detected between-host clusters are less than or equal to the cut-off pairwise patristic distance, then increase the percentile threshold and identify the new set of between-host clusters. If the median pairwise patristic distance of any of the clusters is above the cut-off pairwise patristic distance then regard the previous threshold (current threshold - 0.001) as the optimal clustering threshold.
6. Identify between-host clusters detected using the optimal clustering threshold (identified in 5) as likely clusters of recent HCV transmission.

The pairwise patristic distances that allowed detection of between-host clusters was examined starting at 0.001 and ending at 0.48 for gt1 and 0.5 for gt3, respectively. The lower value was defined as the minimum percentile threshold where only within-host clusters were detected, while the upper values were defined as the maximum percentile thresholds where all sequences were included in a single between-host cluster (Figure 2 in main article text). The optimal cut-off patristic distance representing recent transmission clusters was determined firstly by consideration of longitudinally collected within-host sequences representing a measure of the rate of within-host diversification of HCV genomes. The maximum pairwise patristic distances calculated among within-host sequences was 0.099 for gt1 and 0.095 gt3; hence these values were utilised as the cut-off for designation of between-host clusters.

Results

1: Analysis of a single source outbreak of HCV transmission.

The evolution of genetic diversity that arises over time from a single source outbreak was also examined. To do this, publicly available consensus sequences from a cohort of Irish women (n=10) infected with gt1b HCV from a single donor via blood transfusion was utilised (*3*). One consensus sequence was obtained from the source in 1977, and one consensus sequence was

obtained from each of the ten recipients at two later timepoints, in 1996 and 2000. A phylogenetic tree was generated using the E1-HVR1 sequences including both the infected recipients and the source. The pairwise patristic distances was measured between all sequences from the resulting tree and portrayed in relation to the time interval between the sampling time points.

Pairwise patristic distances between Core-NS3 (thus including E1-HVR1) sequences from longitudinal samples of the Irish cohort were obtained via phylogenetic analysis (Technical Appendix Figure 1 A). The pairwise patristic distance between the source and recipient gt1b sequence pairs collected 19 years after the transmission events ranged up to 0.30 (median: 0.018), and was up to 0.027 (median: 0.021) over a 23-year gap (Technical Appendix Figure 1 B). The patristic distance between within-host sequence pairs from the infected recipients in the Irish cohort reached a maximum of 0.044 (median: 6.11E-03) within a 4-year period, while the patristic distance between any two different recipients in the Irish cohort reached a maximum of 0.049 (median: 0.031) within a 4-year period (Technical Appendix Figure 1 C).

These values indicate that pairwise patristic distances in the Irish cohort were much less than the cut-off pairwise patristic distances identified for gt1 and gt3 in the analysis cohort. It should be noted however, that two between-host sequence pairs (subjects 117 and 461 from transmission Cluster A, and subjects 304 and 357 from transmission Cluster B) exhibited similar patristic distances to those found among the Irish cohort samples.

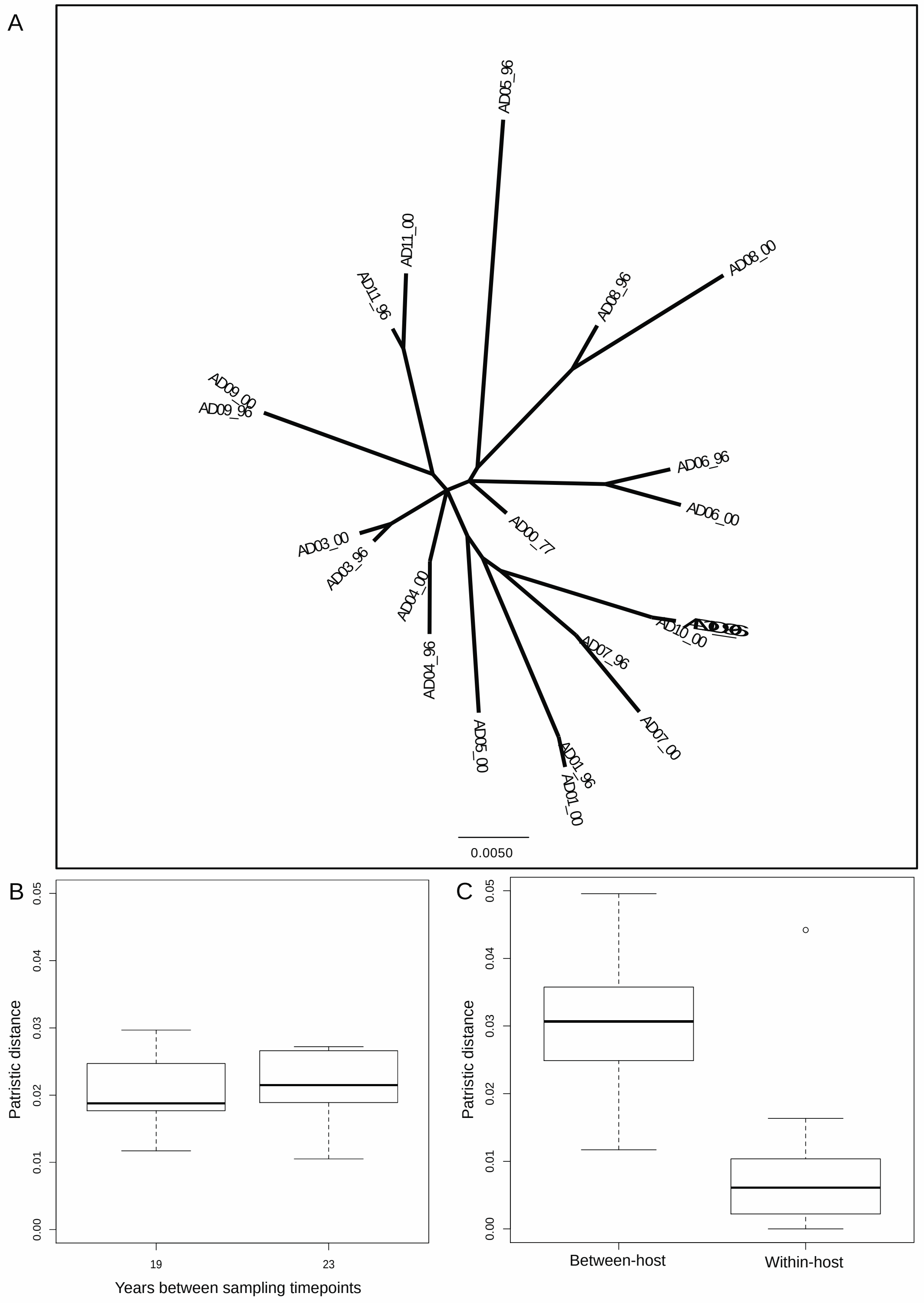

Technical Appendix Figure 1. Phylogenetics and patristic distances analyses of HCV sequences from the Irish cohort. Panel A shows the unrooted phylogenetic tree generated from a maximum likelihood model using a HKY substitution model with gamma distribution. Names on the tips of the tree represent the subject ID followed by the sample collection year. The branch lengths reflect the genetic diversity

between sequences. Within host evolution shows a more closely related viruses when compared to genetic distances between sequences form different recipient. Panel B shows the evolution of the distribution of patristic distances between sequences from the single source and the recipients at two sampling time points (19 and 23 years post-infection). The distribution of patristic distances between the source sequence sampled in 1977 and any recipient sequence sampled in 1996 (19 years apart) ranges from 0.012 to 0.030 while the distribution of patristic distances between the source sequence sampled in 1977 and any recipient sequence sampled in 2000 (23 years apart) ranges from 0.011 to 0.027. Panel C shows the evolution of sequences sampled within host and between hosts in two timepoints (4 years apart). The patristic distance of sequences between two different hosts sampled in 1996 and 2000 ranged from 0.012 to 0.050 while the patristic distance between sequences within-host sampled in 1996 and 2000 ranged from 2.60E-07 to 0.044.

2: Analysis of rare variants from within-host viral quasispecies

The amount of genetic diversity within the quasispecies of a single subject was investigated. This was done to account for potential influence on transmission of a minor viral variant from within the quasispecies of the source to the recipient. An HCV transmission event may plausibly select randomly from any of the circulating variants within the quasispecies to establish the transmitted-founder in the recipient host. Hence, it is feasible that the genetic diversity between a source and a recipient may reflect the maximum diversity within the quasispecies of the source. This was done by analysing deep sequencing data in the E1-HVR1 regions from two subjects (subjects 023 and 240). Sequences of circulating variants at two time points from acute infection until two years post-infection were obtained via next-generation sequencing as described (*4*). From these data, unique HCV variants at a frequency of at least 1% within the viral population (i.e., the quasispecies) were considered for further analysis. This resulted in an average of 15-20 variants in the E1-HVR1 regions per time point. For each subject, a phylogenetic tree was then generated and the pairwise patristic distances between all sequences from the resulting tree were analysed in relation to the time interval between the two sampling time points for each subject.

To address the extent of viral diversity within a single host and the impact of transmission of a ‘diverse’ minor variant, the distribution of pairwise patristic distances between E1-HVR1 variants within the quasispecies in samples collected at two timepoints within one year post-infection from two subjects with primary HCV infections which became chronic (subject 023 and 240). Deep sequencing data were available for these two subjects (*4*), with

frequencies as low as 1% in the viral population. Using these data, separate phylogenetic trees were constructed for the two subjects (Technical Appendix Figure 2 A and B). For subject 023, a maximum patristic distance of 0.033 (median: 0.015) was observed among 7 variants appearing at frequencies between 1% and 36% at 36 days post infection. Meanwhile, a maximum patristic distance of 0.028 (median: 0.013) was observed among 9 variants appearing at frequencies between 1% and 20% at 167 days post infection (Technical Appendix Figure 2 C). Similarly, for subject 240, pairwise patristic distances showed a maximum patristic distance of 0.003 (median: 1.43E-03) among four variants appearing at frequencies between 1% and 69% at 44 days post infection. Meanwhile, a maximum patristic distance of 0.021 (median: 6.8E-03) was observed among 10 variants appearing at frequencies 1% to 42% at 249 days post infection (Technical Appendix Figure 2 D). This result indicates that the maximum genetic distance observed within the host does not exceed the mean genetic distance between consensus sequences identified in between-host analyses.

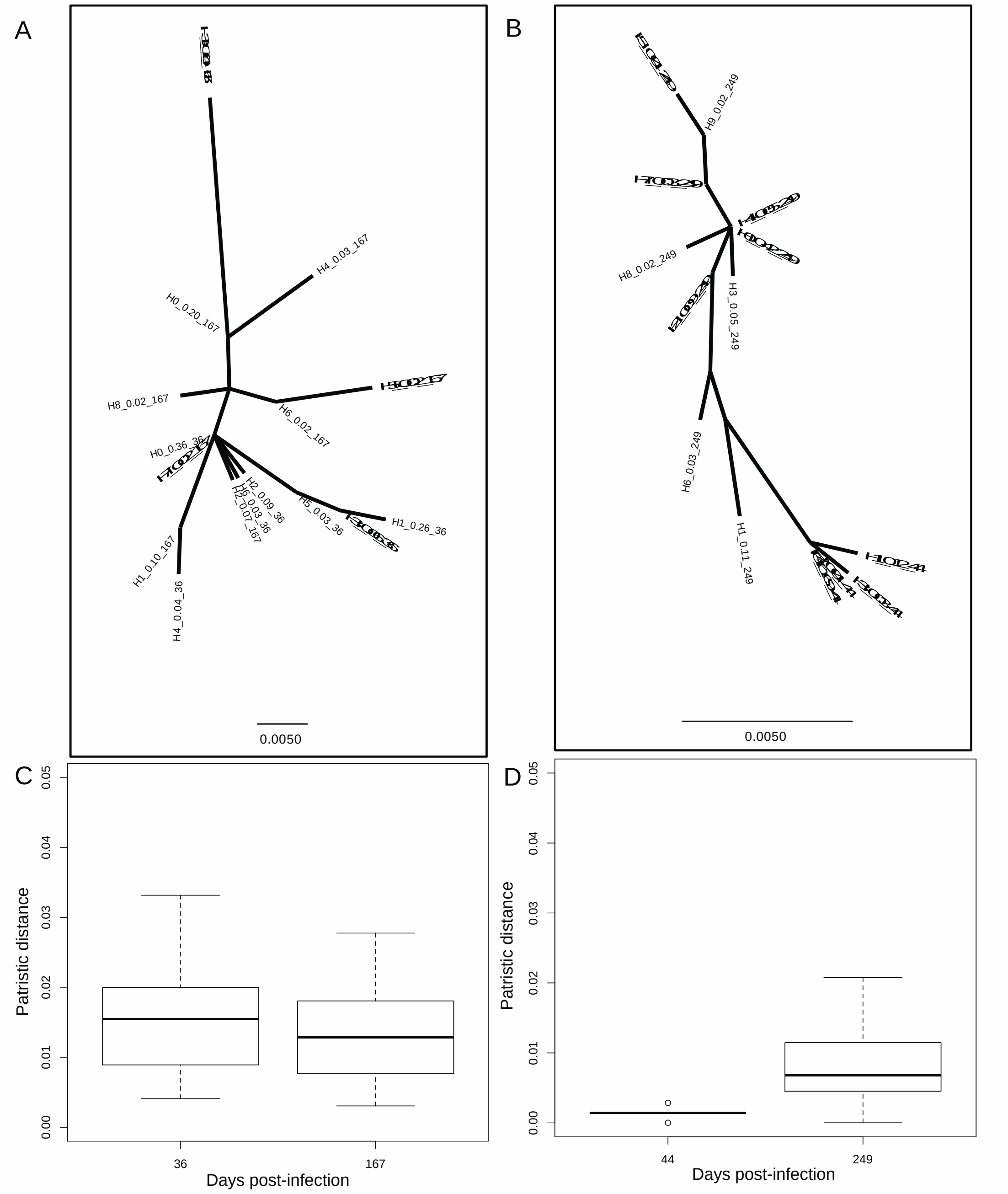

Technical Appendix Figure 2. Analysis of viral quasispecies of two HITS-p subjects (023 and 240) followed longitudinally with deep sequencing analysis of HCV genome. Panel A shows an unrooted phylogenetic tree generated from a maximum likelihood model using a HKY substitution model with gamma distribution on 16 sequences from subject 023. Sequences are obtained from two time points (36 and 167 days post-infection, respectively) representing circulating quasispecies at frequency above 1% in the population. Names on the tips of the tree represent the quasispecies ID followed by the frequency of the quasispecies and the days post-infection. Panel B: phylogenetic tree from 14 sequences from subject 240 obtained from two time points (44 and 249 days post-infection, respectively). Panel C, and D depict

the distribution of pairwise patristic distance between variants in the viral quasispecies at each time-points for subjects 023 and 240, respectively. In subject 023 the distribution of patristic distances ranges from 0.004 to 0.033 at an estimated 36 days since infection, and from 0.003 to 0.028 at an estimated 167 days since infection. For subject 240 (Panel D) the distribution of patristic distances between the variants in the viral quasispecies ranges from 2.30E-07 to 0.003 after an estimated 44 days since infection and from 3.80E-07 to 0.021 after an estimated 249 days since infection.

3. Examination of between-host clusters detected above the optimal cut-off

Two more clusters were detected just above the selected patristic distance thresholds. A putative between-host cluster was detected in the gt1 data, containing sequences from subjects 247 and 418 (designated as Cluster D, Figure 1 in main article text), with a median pairwise patristic distance of 0.149 (0.05 above the cut-off). Similarly in the gt3 data, a putative between-host cluster was detected consisting of sequences from subjects 089 and 082 (designated as Cluster E, Figure 1 in main article text) with a mean patristic distance of 0.246 (0.051 above the cut-off). No prison co-location episodes were found for the two subjects in Cluster D (Technical Appendix Table 1). For putative cluster E, subject 082 was identified as a possible source of transmission and was estimated to have become viremic with gt3 on June 09, 2006. Subject 082 was co-located with subject 089 in a prison for 14 days (August 27 until September 4, 2006), but denied injecting and sharing of injecting equipment during the period of co-location, while subject 089 reported otherwise. An estimated 12 months after co-locating with subject 082, subject 089 was found to be viremic with HCV gt3.

Technical Appendix Table 1. Between-host clusters appearing above the optimal threshold

Cluster	Transmission	Period of co-location	Prison	ID	Est. date of infection	Genotype	Sex	ATSI[a]	Continuously in prison[b]	IDU[c]	Equipment sharing[d]	OST[e]	Heroin[f]
D	247 → 418	No co-location	N/A	247	17/11/06	1a	M	Yes	No	No	No	No	Yes
				418	14/01/08	1a	F	No	Yes	No	No	Met [a]	No
E	082 → 089	27/08/06 - 04/09/06	AD	082	09/06/06	3a	M	No	Yes	No	No	No	No
				089	29/09/07	3a	M	No	Yes	Yes	Yes	No	No

[a] Aboriginal and/or Torres Strait Islander descent. [b] Continuously in prison 6 months prior to estimated date of infection. [c] Injecting drug use during the period of co-location. [d] Sharing injecting equipment during the co-location period. [e] Opioid substitution therapy during the period of co-location. [f] Injecting heroin during the period of co-location.

Technical Appendix Table 2. Distribution of movements between prison locations and release from prison to the outside community of subjects from the HITS-p cohort during the study period

Group	Movement	Min	Max	Mean	Median	Standard deviation	25th percentile	75th percentile
HITS-p	Movements [a]	1	65	17.22	14	12.10	8	24.50
(n=498)	Transfers [b]	1	56	13.98	11	10.47	6	19
	Release [c]	0	15	3.44	3	2.46	2	5
Uninfected	Movements	1	62	14.85	12	10.78	6	22
(n=317)	Transfers	1	50	12.14	10	9.44	5	18.25
	Release	0	15	2.94	2	2.08	2	4
Total incident cases	Movements	2	65	21.44	19	13.11	12	30
(n=181)	Transfers	2	56	17.25	16	11.36	9	23
	Release	0	14	4.32	4	2.82	2	6
Incident cases excluded	Movements	2	65	21.01	19	12.80	11.25	29
(n=102)	Transfers	2	55	16.99	16	11	9	23
	Release	0	14	4.18	3	2.82	2	`6
Study cohort	Movements	3	63	22.01	18	13.55	12	30
(n=79)	Transfers	2	56	14	17.59	11.89	8.25	24
	Release	0	14	4.50	4	2.83	2.25	7
Cluster members	Movements	12	58	28.86	30	15.75	17	34
(n=7)	Transfers	8	45	23	22	12.70	14.5	28.5
	Release	1	13	5.86	4	3.89	4	7.50

[a] Movements across different prisons excluding prison visits less than 24 hours and including release to the outside community. [b] Transfers from one prison to another prison. [c] Release from prison to the outside community.

References

1. Prosperi MC, Ciccozzi M, Fanti I, Saladini F, Pecorari M, Borghi V, et al. A novel methodology for large-scale phylogeny partition. Nat Commun. 2011;2:321. PubMed http://dx.doi.org/10.1038/ncomms1325

2. Fourment M, Gibbs MJ. PATRISTIC: A program for calculating patristic distances and graphically comparing the components of genetic change. BMC Evol Biol. 2006;6:1. PubMed http://dx.doi.org/10.1186/1471-2148-6-1

3. Bailey JR, Laskey S, Wasilewski LN, Munshaw S, Fanning LJ, Kenny-Walsh E, et al. Constraints on viral evolution during chronic hepatitis C virus infection arising from a common-source exposure. J Virol. 2012;86:12582–90. PubMed http://dx.doi.org/10.1128/JVI.01440-12

4. Bull RA, Luciani F, McElroy K, Gaudieri S, Pham ST, Chopra A, et al. Sequential bottlenecks drive viral evolution in early acute hepatitis C virus infection. PLoS Pathog. 2011;7:e1002243. PubMed http://dx.doi.org/10.1371/journal.ppat.1002243
